# Risk assessment and bioburden evaluation of *Agrobacterium tumefaciens*-mediated transient protein expression in plants using the CaMV35S promoter

**DOI:** 10.1186/s12896-023-00782-w

**Published:** 2023-06-07

**Authors:** Matthias Knödler, Paul Winman Reunious, Johannes Felix Buyel

**Affiliations:** 1 Fraunhofer Institute for Molecular Biology and Applied Ecology IME, Forckenbeckstrasse 6, 52074 Aachen, Germany; 2 Institute for Molecular Biotechnology, RWTH Aachen University, Worringerweg 1, 52074 Aachen, Germany; 3 Department of Biotechnology (DBT), Institute of Bioprocess Science and Engineering (IBSE), University of Natural Resources and Life Sciences, Vienna (BOKU), Muthgasse 18, 1190 Vienna, Austria

**Keywords:** Plant molecular farming, Process biosafety, Product toxicity, Promoter activity, *Rhizobium radiobacter*

## Abstract

Large-scale transient expression of recombinant proteins in plants is increasingly used and requires the multi-liter cultivation of *Agrobacterium tumefaciens* transformed with an expression vector, which is often cloned in *Escherichia coli* first. Depending on the promoter, unintentional activity can occur in both bacteria, which could pose a safety risk to the environment and operators if the protein is toxic. To assess the risk associated with transient expression, we first tested expression vectors containing the CaMV35S promoter known to be active in plants and bacteria, along with controls to measure the accumulation of the corresponding recombinant proteins. We found that, in both bacteria, even the stable model protein DsRed accumulated at levels near the detection limit of the sandwich ELISA (3.8 µg L^−1^). Higher levels were detected in short cultivations (< 12 h) but never exceeded 10 µg L^−1^. We determined the abundance of *A. tumefaciens* throughout the process, including infiltration. We detected few bacteria in the clarified extract and found none after blanching. Finally, we combined protein accumulation and bacterial abundance data with the known effects of toxic proteins to estimate critical exposures for operators. We found that unintended toxin production in bacteria is negligible. Furthermore, the intravenous uptake of multiple milliliters of fermentation broth or infiltration suspension would be required to reach acute toxicity even when handling the most toxic products (LD_50_ ~ 1 ng kg^−1^). The unintentional uptake of such quantities is unlikely and we therefore regard transient expression as safe in terms of the bacterial handling procedure.

**Supplementary Information:**

The online version contains supplementary material available at 10.1186/s12896-023-00782-w.

## Introduction

Plants have been used for the production of valuable small molecules and recombinant proteins since the early 1980s, and this has evolved from a niche topic to a mature technology that now competes with the dominant platforms based on microbial and mammalian cells [1–4]. Plants have several advantages over cell-based systems, including their inability to support the replication of human viruses, the ease of process scale-up, and short process development times [1, 5, 6]. The latter is often achieved by transient expression, which requires a scalable gene delivery system for each batch of plants [7]. DNA transfer is often mediated by the bacterium *Agrobacterium tumefaciens* (*Rhizobium radiobacter*) in species such as *Nicotiana benthamiana*, tobacco, Arabidopsis and lettuce [8–13]. The expression vector is generally cloned in *Escherichia coli*, so transient expression requires the transformation of *E. coli* and *A. tumefaciens*, resulting in two different genetically modified organisms (GMOs) [14]. The use of GMOs requires a formal risk assessment to quantify the hazard posed to personnel and the environment, and the likelihood of harm in practice [5, 15]. Based on this assessment, GMOs are categorized and the precautions required for handling are defined, typically using four biosafety levels (BSLs), where BSL-1 encompasses non-pathogenic GMOs and BSL-2 covers agents with a moderate potential hazard [16–19]. The BSL rating is linked to the type and quantity of recombinant protein produced in a GMO. For example, *E. coli* or *A. tumefaciens* strains carrying a plant expression vector encoding a recombinant toxin may be regarded as BSL-2 by the regulatory authorities because promoters from plant viruses, such as the commonly used cauliflower mosaic virus 35S (CaMV35S) promoter [2, 20, 21], are transcriptionally active in bacteria [22–27]. However, the risk assessment routines set out in international guidelines (e.g., the World Health Organization’s Laboratory Biosafety Manual [28]) and supranational laws (e.g., European Union Directive 2000/54/EC [29]) may be interpreted differently at the national level [30]. Accordingly, the same GMO may receive different BSL ratings depending on the country of assessment. This can introduce uncertainty when planning large-scale production, because investment costs for equipment such as filters [31, 32], and especially infrastructure, increase by as much as 25% when BSL-2 is required instead of BSL-1 [33]. Harmonized regulations and classifications would help to resolve this situation [34].

**Fig. 1:**
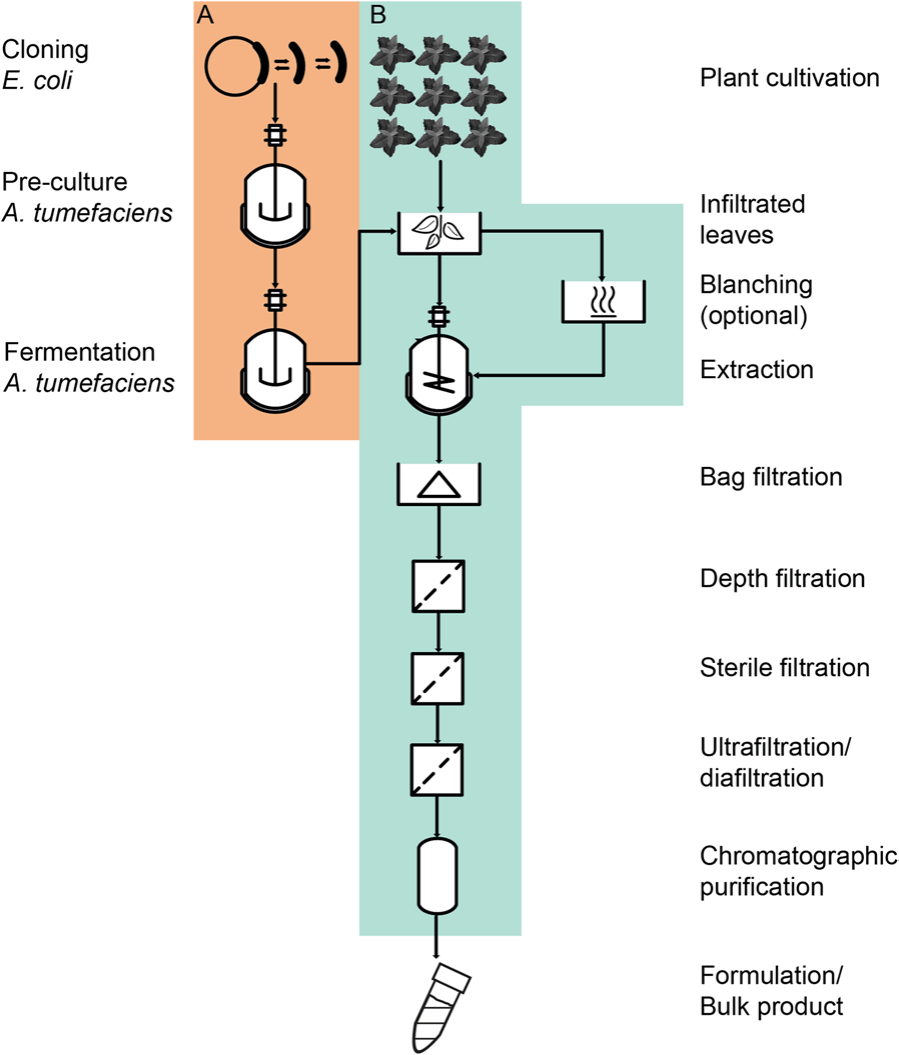
Schematic process flow of *A. tumefaciens*-mediated transient expression in plants. **A** Cloning in *E. coli*, *A. tumefaciens* pre-culture and fermentation for subsequent infiltration of plants. **B** Cultivation of wild-type plants, followed by infiltration with the *A. tumefaciens* suspension and incubation to facilitate transient expression with an optional blanching step before extraction. Downstream processing starts with plant biomass extraction, and includes clarification (e.g., depth filtration), an optional UF/DF step, and purification by chromatography

Although plant promoters show some transcriptional activity in bacteria, and *A. tumefaciens* can persist in transgenic plants grown in greenhouses [35], it is currently unclear how the bacteria spread to the environment from laboratory equipment and surfaces. Also, little is known about the resulting protein accumulation in bacteria and the *A. tumefaciens* bioburden during transient expression in plants (Fig. 1), especially during infiltration and extraction, which would facilitate evidence-based risk assessment. We therefore set out to quantify recombinant protein accumulation resulting from double enhanced CaMV35S promoter activity in *A. tumefaciens* and *E. coli* under representative cultivation conditions compared to bacterial reference promoters. We also monitored the *A. tumefaciens* bioburden during the preparation and processing of infiltrated plant biomass, including an optional blanching step that facilitates subsequent product purification [36–38]. We combined these results with worst-case protein toxicity data and unintended intravenous delivery to personnel in order to derive volumes of process intermediates necessary to reach a hypothetical median lethal dose (LD_50_). Our results will facilitate the development of safety measures that match actual risks during relevant process steps, taking into account recombinant protein expression levels and activities as well as the colonizing capacity of GMOs, as described, for example, in Annex 1 of the German genetic engineering safety enactment (Gentechnik-Sicherheitsverordnung, GenTSV) [39].

## Materials and methods

### Molecular cloning and plant expression vectors

The CaMV35S promoter driving the expression of DsRed, a red fluorescent protein from *Discosoma* sp., and both chains of a monoclonal IgG1 antibody in the plant expression vector pTRAc [40] was replaced with the β-lactamase (*bla*) promoter from the *Tn*3 transposon [41], or the T7 promoter from bacteriophage T7 RNA polymerase [42] (Additional file 1: Table S1) in a restriction-ligation reaction using AscI, EcoRI and NheI (New England BioLabs, Ipswich, USA). Promoter sequences with compatible ends were generated by PCR using specific primers (Additional file 1: Table S2). DNA was purified using a NucleoSpin kit (Machery-Nagel, Düren, Germany) and ligated using T4 DNA ligase (New England BioLabs). The resulting plasmids (Additional file 1: Table S3) were introduced into chemically competent *E. coli* DH5α cells and electro-competent *A. tumefaciens* GV3101 cells after purification.

### Sources of plant seeds and cells

*N. tabacum* L. cv Bright Yellow-2 cells were obtained from the Leibniz-Institute DSMZ Deutsche Samlung von Mikroorganismen und Zellkulturen GmbH on the 20th of January 2020 (ID PC 1181). Seeds from *N. benthamiana* plants were a donation from the RWTH Aachen University in 2006.

### Bacterial cell culture and extraction

For *E. coli* DH5*α*, we inoculated 0.3 L of sterile lysogeny broth (LB) medium (5 g L^−1^ yeast extract, 10 g L^−1^ tryptone, 0.5 g L^−1^ sodium chloride, supplemented with 100 mg L^−1^ ampicillin) with 0.3 mL of an overnight pre-culture in a 2-L non-baffled Erlenmeyer flask. The cultures were incubated for up to 48 h at 37 °C, shaking at 180 rpm. We took 50-mL samples after 12, 24 and 36 h for protein analysis.

For *A. tumefaciens* GV3101:pMP90RK, we inoculated 0.3 L of PAM4 medium (20 g L^−1^ soy peptone, 0.5 L^−1^ yeast extract, 5.0 g L^−1^ fructose, 1.0 g L^−1^ magnesium sulfate, pH 7.0) [43] or yeast extract broth (YEB) medium (5.0 g L^−1^ beef extract, 1.0 g L^−1^ yeast extract, 5.0 g L^−1^ soy peptone, 5.0 g L^−1^ sucrose, 0.5 g L^−1^ magnesium sulfate, pH 7.0), each supplemented with 25 mg L^−1^ kanamycin, 25 mg L^−1^ rifampicin and 50 mg L^−1^ carbenicillin, with 0.3 mL of an overnight pre-culture in a non-baffled 2-L Erlenmeyer flask. The cultures were incubated for up to 100 h at 26 °C, shaking at 160 rpm. Wild-type *A. tumefaciens* controls were cultured under the same conditions but without antibiotics. We took 50-mL samples after 24, 48 and 72 h to determine the optical density at 600 nm (OD_600nm_) and the Boltzmann model (Eq. 1) was fitted to the data.

1 $$f\left( x \right) = \frac{{A_{1} - A_{2} }}{{1 + e^{{\left( {x - x_{0} } \right)/dx}} }} + A_{2}$$

where *A*_*1*_ is the initial (OD_600nm_) value, *A*_*2*_ is the final (OD_600nm_) value, *x* is the cultivation time, *x*_*0*_ is the curve center, *dx* is the time constant, and *f*(*x*) is the OD_600nm_ as a function of time.

Harvested culture samples were also centrifuged (4000×*g*, 10 min, 10 °C) and pellets were homogenized on ice using a PowerGen 125 IKA Ultra Turrax homogenizer (Thermo Fisher Scientific, Waltham, USA) in 5 mL NEBExpress Gram-negative bacteria extraction buffer (New England BioLabs). We applied three pulses of 1 min with pauses of 2 min between. Extracts were clarified by centrifugation (4000×*g*, 10 min, 10 °C) and passage through a 0.2 µm MiniSart syringe filter (Sartorius, Göttingen, Germany) before protein analysis.

### Transient expression in differentiated N. benthamiana plants

Seeds were germinated on stone wool blocks soaked with 1.0 g L^−1^ Ferty 2 Mega fertilizer solution (Planta Düngemittel, Regenstauf) and incubated for ~ 7 days before reducing the number of germ buds to one per block. The stone wool blocks were placed into custom-made plastic trails and incubated in a greenhouse (25/22 °C day night temperature, ~ 14-h photoperiod, ~ 70% relative humidity). Natural light was augmented if necessary with 400-W IP65 or SON-k sodium discharge lamps (Phillips, Amsterdam, Netherlands) [31, 37]. The plants were irrigated with 1.0 g L^−1^ fertilizer solution for 12 min 2–4 times per day using an ebb-and-flow hydroponics system that removed residual liquid after each watering phase. *N. benthamiana* plants were infiltrated with *A. tumefaciens* carrying vectors for the expression of DsRed or antibody M12 at 42–49 days after seeding by vacuum infiltration. Specifically, a bucket containing ~ 5 L of OD_600nm_ = 0.5 bacterial suspension was placed in a desiccator and plants were submerged headlong into the liquid. Then, the pressure was reduced to 5 kPa (50 mbar) using a vacuum pump, held at this level for 2 min and abruptly released. Infiltrated plants were briefly drained of residual infiltration suspension for 1 min and prepared for S2-compliant incubation by wrapping them into translucent plastic film. If necessary, additional bacterial suspension was added to the bucket to maintain a level of ~ 5 L and the suspension was manually agitated for ~ 30 s in between each plant infiltration cycle to prevent sedimentation. The infiltration was conducted at a temperature of ~ 21 °C. Infiltrated plants were harvested after incubation for 5 days at 21 °C and ~ 70% relative humidity and were used for acidic blanching [44].

### Acidic blanching of N. benthamiana leaf material

Leaves were blanched in an EKA 3338 heated vessel (Clatronic International, Kempen, Germany) equipped with an MD‐6Z pump (~ 6.0 L min^−1^) to maintain a constant circulation of 20 L blanching buffer (20 mM trisodium citrate, pH 4.0). The temperature was set to 70 or 80 °C before leaves were submerged in the blanching buffer and carefully agitated to ensure uniform blanching. After blanching for up to 15 min, the leaves were transferred to a bucket filled with cold tap water for ~ 15 s and then carefully dried with paper towels before extraction. The apparent gain in leaf mass due to residual blanching buffer was recorded and used to correct any dilution effect during subsequent protein extraction.

### Extraction and filtration

Freshly harvested or blanched *N.* *benthamiana* plant material was homogenized using a ProBlend 6 blender (Phillips, Amsterdam, Netherlands) as previously described [45] and a modified extraction buffer (50 mM sodium phosphate, 500 mM sodium chloride, 10 mM sodium bisulfite, pH 6.0). The homogenate pH was immediately adjusted to 8.0 with 0.4 M trisodium phosphate. Coarse particles were removed by passing the homogenate through an acuraLine BP-420-1 bag filter (Fuhr, Klein-Winternheim, Germany) with a 1-µm nominal pore size. Fine particles were removed by depth filtration using dual V700P and V100P layers with nominal pore sizes of 8–20 and 1–3 µm, respectively. Depth filtration was performed at a constant volumetric flux of ~ 1.0 L m^−2^ min^−1^ using a Masterflex SE peristaltic pump (Masterflex, Gelsenkirchen, Germany) up to a maximum inlet pressure of 0.2 MPa. The depth filtrate was passed through a Sartopore2 150 sterile filter (Sartorius) with a pore size combination of 0.45 and 0.20 µm.

### Bioburden assessment of plant infiltration and plant biomass processing

Process samples (Table 1) were plated on YEB agar (1.5% m v^−1^) containing the same antibiotics as above and were incubated at 28 °C and ~ 25% relative humidity. Colony forming units (CFU) were counted by visual inspection every 24 h up to 5 days after plating. Specifically, the top and bottom of infiltrated, blanched and wild-type control *N. benthamiana* leaves were tested by direct contact with a contact area of ~ 0.0056 m^2^ per leaf (~ 80% of the total area of a 94 mm diameter Petri dish). Up to 250 mL of the blanching buffer (after processing wild-type control or infiltrated leaves) was also passed through a 0.45-µm filter and the filter cake was used in the direct contact test with a contact area of ~ 0.0017 m^2^. Furthermore, 15-mL samples were taken from wild-type control and infiltrated leaves before and after blanching as well as after bag, depth and sterile filtration. One set of aliquots from these samples was incubated for 15 min in 15-mL reaction tubes to allow dispersed particles to sediment and a second set was centrifuged (15,000×g, 1 min, 21 °C). We then plated 0.1 mL of each supernatant undiluted and also as 1:100 and 1:1000 dilutions in PBS. Finally, air samples during infiltration as well as during blanching were taken at three representative positions 300–600 mm from each device (Additional file 1: Fig. S1) using an exposure time of 15 min. For these samples, we used both selective and antibiotic-free YEB agar plates.

**Table 1:** Process steps during transient expression in *N. benthamiana* and monitoring measures to detect *A. tumefaciens*

Process step (–)	Sample type (–)	YEB medium (–)	Unit
Infiltration	Air sample	Selective/non-selective	CFU m^−2^
Incubation	Direct contact test	Selective	CFU m^−2^
Blanching	Air sample	Selective/non-selective	CFU m^−2^
Extraction	Liquid	Selective	CFU L^−1^
Bag filtration	Liquid	Selective	CFU L^−1^
Depth filtration	Liquid	Selective	CFU L^−1^
Sterile filtration	Liquid	Selective	CFU L^−1^

We sampled wild-type control, infiltrated as well as infiltrated and blanched plant biomass

*CFU* colony forming units, *YEB* yeast extract beef medium

All samples were collected across a set of seven independent experiments using up to six replicate plates per sample per batch (including dilutions). Given the log-normal distribution of bacterial population counts on leaf surfaces [46], we used the geometric mean (Eq. 2) and its standard deviation (Eq. 3), which better represent such distributions than their arithmetic counterparts [47], despite some debate about the standard deviation formula [48, 49].

2 $$\overline{x}^{*} = exp\left( {\frac{1}{n}\mathop \sum \limits_{i = 1}^{n} {\text{log}}\left( {x_{i} } \right)} \right) = \left( {\mathop \prod \limits_{i = 1}^{n} x_{i} } \right)^{\frac{1}{n}}$$

3 $$s^{*} = exp\left( {\left[ {\frac{1}{n - 1}\mathop \sum \limits_{i = 1}^{n} \left[ {{\text{log}}\left( {\frac{{x_{i} }}{{\overline{x}^{*} }}} \right)} \right]^{2} } \right]^{\frac{1}{2}} } \right)$$

where $\overline{x}^{*}$ is the mean of the log-normal distributed data (i.e., the geometric mean), *n* is the sample size, *x* is the value (here CFU count) of observation *i*, and s* is the geometric standard deviation. CFU counts were converted to an OD_600nm_ using Eq. 4 [50].

4 $$OD_{{600\,{\text{nm}}}} \left[ - \right] = CFU \left[ {\frac{1}{{{\text{mL}}}}} \right] \times 1.43 \times 10^{ - 9}$$

For air and direct contact samples, the CFU count per square meter was converted to a volumetric equivalent assuming that a liquid level of ~ 1 mm height (1.0 L m^−2^) would be needed to re-suspend the bacteria.

### Protein expression in plant cell packs

Tobacco (*Nicotiana tabacum*) Bright Yellow-2 (BY-2) cells from a continuous culture were expanded in 0.5-L shake flasks for 5 days to a cell wet mass of 200 g L^−1^ in an ISFX-1 shaker (Adolf Kühner, Birsfelden, Switzerland) at 160 rpm and 26 °C, and were used for the preparation of plant cell packs (PCPs) as previously described [51]. *Agrobacterium tumefaciens* cultures were adjusted to an OD_600nm_ of 0.5 using infiltration buffer (0.5 g L^−1^ Murashige-Skoog salt M0221, 50.0 g L^−1^ sucrose, 2.0 g L^−1^ glucose monohydrate, 0.04 g L^−1^ acetosyringone, pH 5.6) before application to the PCPs [51]. After incubation in the dark at 26 °C and 80% relative humidity for 4 days, the PCPs were harvested for homogenization in an MM300 bead-mill (Retsch, Haan, Germany). We added three volumes (v m^−1^) of extraction buffer (20 mM sodium phosphate, 500 mM sodium chloride, 10 mM sodium bisulfite, pH 8.0) followed by three 30-s pulses at 28 Hz. After centrifugation (4000×*g*, 8 min, 10 °C) the supernatant was analyzed or stored at − 20 °C.

### Protein quantification

The samples were analyzed by LDS-PAGE [31] and western blotting [52] as previously described. DsRed was detected using a monoclonal rabbit anti-His_6_ primary antibody and an alkaline phosphatase (AP)-labeled goat anti-rabbit secondary antibody, whereas M12 was detected using AP-labeled polyclonal goat anti-human heavy and light chain antibodies. DsRed in PCP extracts was quantified by fluorimetry [31].

For sandwich ELISA, the wells of high-binding 96-well ELISA plates (Greiner Bio-One, Kremsmünster, Austria) were coated with 100 µL of the appropriate capture antibody prepared in coating buffer (14.3 mM disodium carbonate, 34.9 mM sodium bicarbonate, pH 9.6) at a concentration of 0.2 mg L^−1^. After incubation for 18 h at 4 °C, the coating solution was removed and the wells were washed with blocking buffer (50 g L^−1^ skimmed milk powder in PBST: 137 mM sodium chloride, 2.7 mM potassium chloride, 10 mM disodium hydrogen phosphate, 1.8 mM sodium dihydrogen phosphate, 0.05% (v v^−1^) Tween-20, pH 7.2) for 1 h at 21 °C. Excess liquid was removed and the coated plates were directly used for ELISA experiments.

Extracts and dilution series of standards (purified target proteins) were prepared in blocking buffer (50 g L^−1^ skimmed milk powder in PBST) before pipetting 100 µL into the coated wells as technical triplicates or duplicates, respectively. After incubation for 1 h at 21 °C on a shaker at 10 rpm, the liquid was removed and wells were washed five times with 250 µL wash buffer (50 mM Tris, 15 mM sodium chloride, pH 7.2). The detection antibody was prepared in blocking buffer using concentrations as recommended by the manufacturer (1.5–2.5 g L^−1^) and 100 µL was added to each well. After incubation for 2 h at 21 °C and five wash cycles with 250 µL wash buffer, we added 100 µL 1-Step PNPP solution (Thermo Fisher Scientific) per well and mixed thoroughly by agitation at 10 rpm. The plate was incubated for 30 min at 21 °C before stopping the reaction by adding 50 µL 2.0 M sodium hydroxide to each well and measuring the absorbance at 405 nm in duplicate using a Synergy H1 microplate reader (BioTek, Winooski, USA) at 21 °C. The absorbance of eight standards of DsRed-His_6_ or M12 IgG1 [31, 38] in the range 0.003–300 mg L^−1^ was measured in triplicate. Blank-corrected extract samples were log transformed, and a linear log–log curve fit (R^2^ = 0.98) was used to calculate recombinant protein concentrations. We used a wet mass to dry mass conversion factor of 0.2 [53, 54] combined with an OD_600nm_ to dry mass conversion factor of 0.396 g L^−1^ (*E. coli*) or 0.409 g L^−1^ (*A. tumefaciens*) [55, 56] to calculate cell mass-specific recombinant protein concentrations.

## Results and discussion

### CaMV35S promoter activity in bacterial cells

**Fig. 2:**
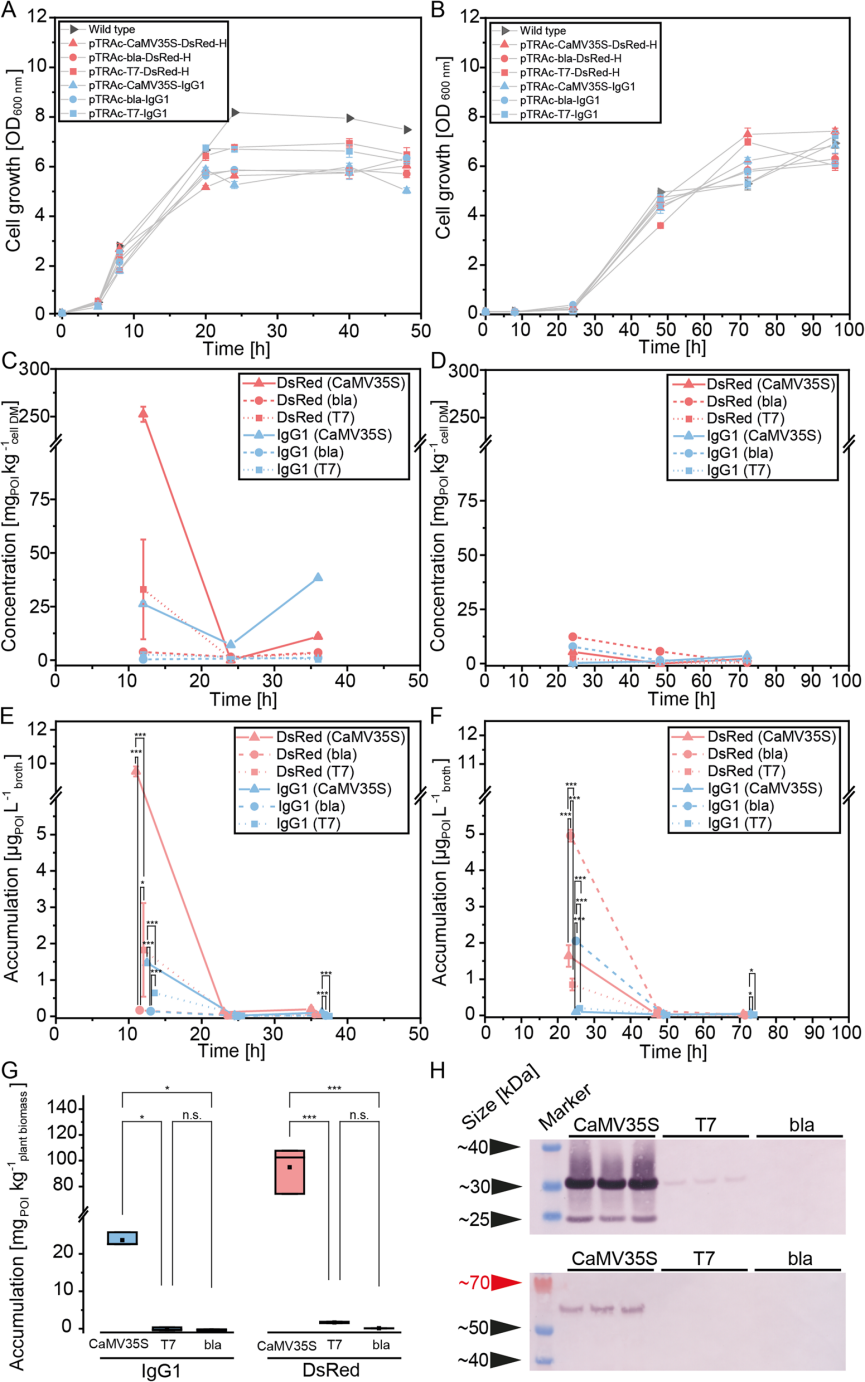
Evaluation of bacterial growth and recombinant protein accumulation. Growth curves of *E. coli* (**A**) and *A. tumefaciens* (**B**) carrying pTRAc vectors (Additional file 1: Table S3) for the expression of DsRed or IgG1 under control of different promoters. DsRed and IgG1 concentrations in *E. coli* (**C**) and *A. tumefaciens* (**D**) per cell dry mass were determined by ELISA. Cell-mass specific recombinant protein concentrations were calculated using a dry mass to OD_600nm_ conversion factor of 0.396 g^−1^ (*E. coli*) and 0.409 g.^−1^ (*A. tumefaciens*) [55, 56] and a wet mass to dry mass conversion factor of 0.2. DsRed and IgG1 concentration in *E. coli* (**E**) and *A. tumefaciens* (**F**) fermentation broth were quantified by ELISA. The sampling time points were shifted by ± 1.5 h to display significant differences between samples. IgG1 and DsRed accumulation levels in PCPs (**G**) using different promoters. Western blot of triplicate PCP extracts (**H**) using plasmids with different promoters for the expression of DsRed (top row) and IgG1 (bottom row). DsRed was detected using a rabbit anti-His_6_ primary antibody and an alkaline phosphatase (AP)-labeled goat anti-rabbit secondary antibody, whereas IgG1 was detected using an AP-labeled goat anti-human antibody. Full-size blots can be found as Additional file 2: Fig. S2 “Risk_and_bioburden_knödler_et_al_full-size_blots_v2”. CaMV35S—double-enhanced cauliflower mosaic virus 35S promoter with strong activity in plants; *bla*—β-lactamase promoter with activity in bacteria; T7—bacteriophage T7 promoter with minimal activity in bacteria unless the corresponding polymerase is expressed. Data are means ± SD (n = 3 biological replicates, two-way ANOVA with Bonferroni correction and a significance threshold of α = 0.05; *p ≤ 0.05, **p ≤ 0.01, ***p ≤ 0.001)

The growth of *E. coli* and *A. tumefaciens* strains transformed with pTRAc for DsRed and IgG1 expression (Additional file 1: Table S3) was similar to that of untransformed controls (Fig. 1A, B). *E. coli* grew significantly faster than *A. tumefaciens* when comparing the *x*_*0*_ coefficients of a Boltzmann model (Eq. 1) fitted to the growth curve data (two-sided two-sample t-test, *p* < 0.001), which was anticipated because the doubling times are ~ 0.3 and ~ 3 h, respectively [57, 58]. Whereas all *A. tumefaciens* cultures reached a similar maximum OD_600nm_ of ~ 6.8 ± 0.5 (± SD, n = 7), the transformed *E. coli* clones reached a significantly lower maximum OD_600nm_ (*p* < 0.001) of 6.5 ± 0.8 (± SD, n = 6) after 20 h compared to ~ 7.7 ± 0.1 (± SD, n = 3) for the control (Fig. 2A). This may reflect the plasmid-associated metabolic burden in *E. coli* [59, 60] because pTRAc vectors have a higher copy number in this species than in *A. tumefaciens* [61–63] (Fig. 2B).

The concentrations of DsRed and IgG1 in *E. coli* and *A. tumefaciens* extracts were below the western blot detection limit (0.27 mg L^−1^ for DsRed and 2.60 mg L^−1^ for IgG1). The more sensitive ELISA revealed concentrations as a proportion of cell dry mass of up to ~ 250 mg kg^−1^ in *E. coli* and 12 mg kg^−1^ in *A. tumefaciens* (Fig. 2C, D). This corresponded to concentrations below 10 µg L^−1^ for DsRed and 2 µg L^−1^ for IgG1 in *E. coli* fermentation broth or below 3 µg L^−1^ for both proteins in *A. tumefaciens* cultures (Fig. 2E, F). Interestingly, and despite their stability [64–66], both proteins were only present at relevant levels during the lag and early log phases and were barely detectable after 24 h regardless of the protein or species, corresponding to a consistently low protein concentration in the fermentation broth of < 0.3 µg L^−1^. Specifically, DsRed was 5–10 times more abundant than the IgG1, probably due to the greater complexity of immunoglobulins and/or the inability of bacteria to introduce sufficient disulfide bonds to ensure correct antibody folding and stability [67, 68]. However, a similar difference in the accumulation of these proteins has also been observed in transgenic plants [69]. We found that the CaMV35S promoter produced the highest levels of recombinant protein in *E. coli*, but was significantly less active in *A. tumefaciens* for harvests within 24 h (two-sided two sample t-test, *p* < 0.032). We speculate that *A. tumefaciens* may have evolved mechanisms to inhibit transcription initiated from promoters that are active in plants because these bacteria naturally carry plant-specific promoters on their Ti-plasmids [70], and the corresponding proteins are not needed by the bacteria. The leaky activity of plant promoters in *A. tumefaciens* would therefore cause an unnecessary metabolic burden [71]. The low protein levels we observed were also consistent with previous studies reporting nonspecific but marginal activity of plant promoters in Gram-negative bacteria based on mRNA levels [22, 23]. It is currently unclear whether tightly regulated or inducible plant promoters [72, 73] are inactive in *A. tumefaciens* and *E. coli*.

As a control experiment, we also used the same *A. tumefaciens* strain for transient expression in PCPs [74]. We found that constructs containing the plant-specific CaMV35S promoter produced ~ 90 mg kg^−1^ and 24 mg kg^−1^ of DsRed and IgG1, respectively, which was significantly more than the other promoters (Fig. 2G, H, Additional file 2: Fig. S2). This was anticipated based on previous reports [44, 51]. The only non-plant promoter that produced detectable levels of DsRed in PCPs was the T7 promoter.

#### *Agrobacterium tumefaciens* bioburden evaluation for a transient expression process using whole plants

**Fig. 3:**
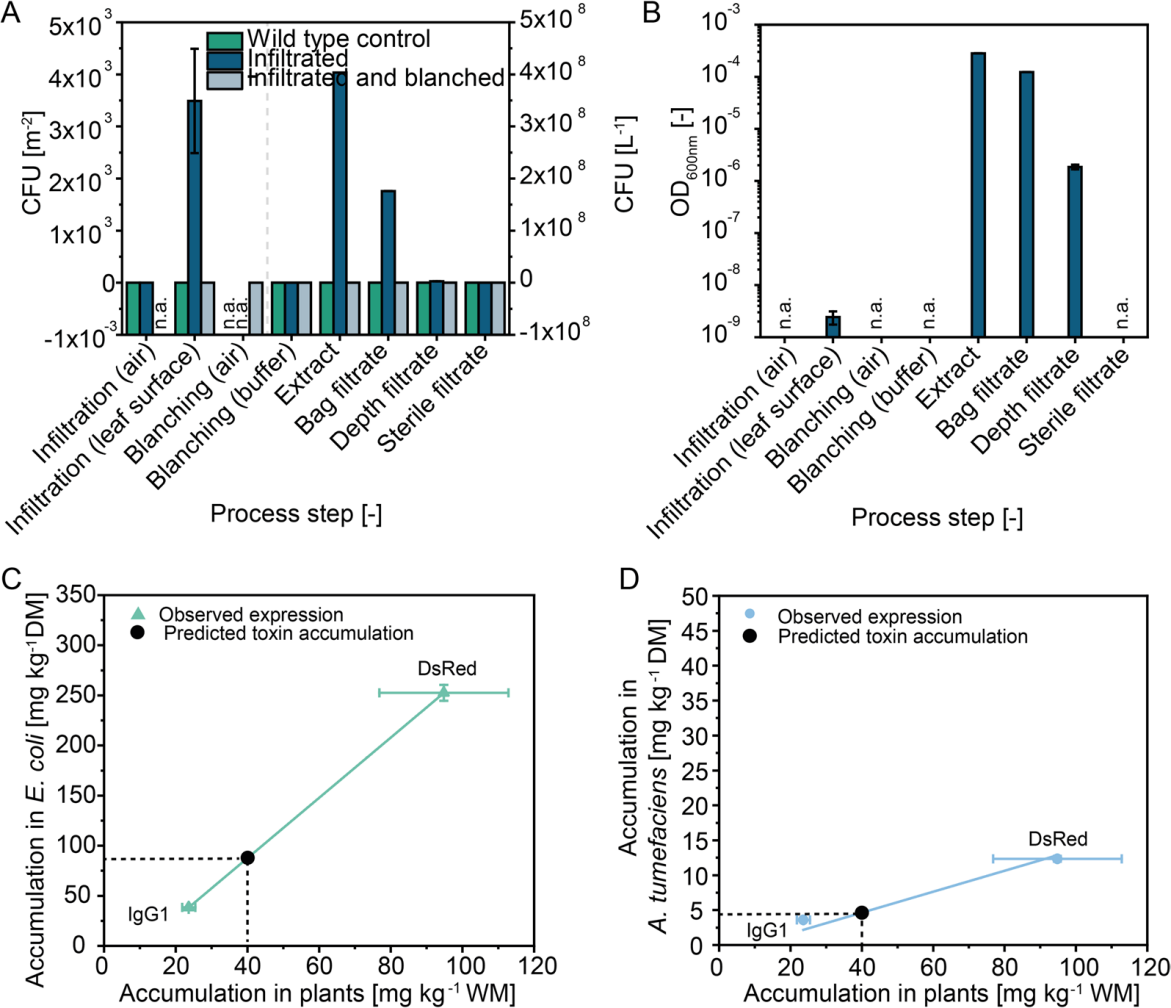
Bioburden and predicted toxin accumulation in bacteria. **A** *A. tumefaciens* bioburden during the infiltration, optional blanching, extraction and clarification of *N. benthamiana* biomass and in the corresponding process intermediates. The CFU per area (air and surface samples) or per liter of process intermediate was assessed on selective and non-selective (air samples only) YEB agar plates. **B** Samples as in **A** but with the CFU [L^−1^] count converted to an equivalent OD_600nm_ [–] using a conversion factor of 6.99 × 10^−13^ [L] based on a published correlation [50]. **C** Correlation between DsRed and IgG1 accumulation in plants and *E. coli* (Pearson’s r = 0.99, R^2^ = 0.99) with an average correlation factor (slope) of 0.43. **D** Correlation between DsRed and IgG1 accumulation in tobacco BY-2 PCPs and *A. tumefaciens* (Pearson’s r = 0.99, R^2^ = 0.96) with an average correlation factor of 0.03. DsRed and IgG1 accumulation in *E. coli* and *A. tumefaciens* were measured by ELISA (Fig. 2C, D). Data are means ± SD (n = 4–21 data points depending on the sample). CFU—colony forming unit; DM—dry mass; n.a.—not applicable; OD—optical density

We monitored the *A. tumefaciens* bioburden during the infiltration of *N. benthamiana* and subsequent incubation, biomass conditioning (blanching), extraction and clarification steps (Table 1). As expected, no *A. tumefaciens* were found at any process step when non-infiltrated wild-type control plants were handled and processed (Fig. 3). We also found no *A. tumefaciens* in any of the air samples during infiltration or blanching, and all blanching buffers were also sterile. The abundance of *A. tumefaciens* on leaf surfaces was ~ 7000 CFU m^−2^ after infiltration but before blanching. Given a typical *N. benthamiana* leaf thickness of 0.25 × 10^−3^ m and a density of 760 kg m^−3^ [75], this corresponded to an *A. tumefaciens* concentration of ~ 40 CFU g^−1^ leaf biomass, which is marginal compared to the 0.3–1.0 × 10^6^ g^−1^ of total epiphytic bacteria found, for example, on rice leaves [76]. Our observation agreed with earlier data suggesting that bacterial communities on tobacco leaves are stabilized by quorum sensing [77], which should prevent the overgrowth of bacterial communities by pathogenic proteobacteria such as *A. tumefaciens* [78]. Furthermore, no *A. tumefaciens* were detected on wild-type or infiltrated leaves after blanching, confirming that this processing method effectively inactivates bacteria before protein extraction. When infiltrated leaves were homogenized without blanching, the *A. tumefaciens* load was ~ 4 × 10^8^ CFU L^−1^ of extract. Given our biomass-to-buffer ratio of 1:3, this corresponded to ~ 10 × 10^6^ CFU g^−1^ fresh leaf mass, which is an order of magnitude more than the concentration calculated above for *A. tumefaciens* on the surface. Finally, the depth filtrates of extracts from infiltrated (but not blanched) leaves revealed that CFU counts were reduced by two orders of magnitude, and no *A. tumefaciens* were detected at all in the sterile filtrates. This indicated that downstream processing steps typically used for plant biomass were able to remove the bacteria effectively, and we concluded that the bioburden of *A. tumefaciens* was small during each process step, especially compared to typical epiphytic bacteria on leaf surfaces.

Interestingly, the abundance of *A. tumefaciens* in laboratories (even those working with plants) is low in relation to other bacteria, with a relative frequency of only ~ 0.001 [79]. Indeed, the same is true overall for the class α-proteobacteria, which accounts for < 1% of all bacteria found in such settings. *A. tumefaciens* is not considered as a laboratory-contaminating reagent introduced by human daily activities, or even a basic environmental bacterium [79]. Furthermore, it is not among the antibiotic-resistant microbes associated with health risks [80]. This bacterium therefore appears to pose little risk to operators in the laboratory even if it is released during transient expression experiments or subsequent processing steps, and is a negligible risk to humans and the environment if it then escapes from the laboratory.

### Risk assessment for the transient expression of toxic proteins in plants

The process steps during which operators can be exposed to relevant quantities of potentially harmful bacteria are limited to fermentation, plant infiltration/incubation, extraction, and the subsequent conditioning and clarification steps up to sterile filtration (Fig. 1). Here, we assumed an intermediate process scale of ~ 200 kg plant biomass as previously discussed [7]. The *E. coli* and *A. tumefaciens* laboratory strains we used are rated as safety class 1, which means they are not harmful to immunocompetent persons [39]. Accordingly, any potential risk associated with their handling arises from the recombinant proteins they produce due to the residual promoter activity in bacteria described above. The risk is highest for toxic proteins with low median lethal doses (LD_50_) for intravenous exposure of 0.001–500 µg kg^−1^ body mass [81–83], with the latter being ~ 80.7 kg for an average person in North America as of 2012 [84]. The risk will also increase in line with the protein concentration, which we estimated by first assuming that protein accumulation in plants and nonspecific expression in bacteria are proportional (e.g., due to target protein stability). We therefore fitted linear models for the CaMV35S-driven accumulation of DsRed and M12 in *N. benthamiana vs E. coli* as well as *N. benthamiana vs A. tumefaciens* (Fig. 3C, D). We then combined these models with the highest levels reported for the transient expression of a recombinant immunotoxin in tobacco PCPs (to remain consistent with the DsRed and IgG1 expression data) of ~ 40 mg kg^−1^ (our unpublished data). This allowed us to estimate the expression of such a protein in both bacterial systems, resulting in values of ~ 80 mg kg^−1^ dry mass in *E. coli* and ~ 4 mg kg^−1^ dry mass in *A. tumefaciens*. Importantly, these values are worst-case scenarios because the correlation with plant-based expression is based on the highest protein accumulation in bacteria, which was only observed at the beginning of cultivation, so the actual toxin concentration when the bacteria are harvested is likely to be lower by one or two orders of magnitude (Fig. 2C–F). Furthermore, the toxin concentration in plants we used to interpolate the corresponding concentration in bacteria was, to our knowledge, the highest reported in PCPs and plant cells so far [21]. Based on these data and our bioburden results, we assessed the risks of an *A. tumefaciens*-mediated transient expression process in plants (Table 2).

**Table 2:** Concentrations of toxic recombinant protein in process fluids due to expression in *A. tumefaciens* and *N. benthamiana* along with the corresponding hazardous process volumes

Process step (–)	Bacteria OD_600nm_ (–)	C_bacteria_ (g DM L^−1^)^b^	C_toxin from bacteria_ (µg L^−1^)^b,c^	C_toxin from plant_ (µg L^−1^)^b,c^	C_total toxin_ [µg L^−1^]^c^	V_critical_ (L) depending on LD_50_ of…			
						500 µg kg^−1^	1 µg kg^−1^	1 ng kg^−1^	V_process_ (L)
Cloning (*E. coli*)	2.00	0.79	62.13	0	62.13	650	1.30	0.001	0.002
Pre-culture (*A. tumefaciens*)	5.00	2.05	8.07	0	8.07	5.00 × 10^3^	10.00	0.01	0.05
Fermentation	10.00	4.09	16.14	0	16.14	2.50 × 10^3^	5.00	0.005	75
Infiltrated leaves^d^	0.50	0.21	0.81	0	0.81	50.00 × 10^3^	100.00	0.10	1500
Extraction^a^	28.20 × 10^−3^/0	11.5 × 10^−3^/0	0.45 × 10^−6^/0	10.10 × 10^3^	10.13 × 10^3^	3.98	7.97 × 10^−3^	7.97 × 10^−6^	800
Bag filtration^a^	12.30 × 10^−3^/0	0.50 × 10^−6^/0	0.19 × 10^−6^/0	9.62 × 10^3^	9.62 × 10^3^	4.19	8.39 × 10^−3^	8.39 × 10^−6^	640
Depth filtration^a^	1.86 × 10^−6^/0	76.1 × 10^−6^/0	3.00 × 10^−6^/0	8.66 × 10^3^	8.66 × 10^3^	4.66	9.32 × 10^−3^	9.32 × 10^−6^	640
Sterile filtration	0	0	0	8.66 × 10^3^	8.66 × 10^3^	4.66	9,32 × 10^−3^	9.32 × 10^−6^	640
UF/DF^e^	0	0	0	0.17 × 10^6^	0.17 × 10^6^	0.23	46.6 × 10^−3^	0.47 × 10^−6^	32

^a^In these steps, the toxic recombinant protein is present due to (unintended) expression in bacteria and deliberate expression in plants

^b^Values correspond to a process without/with blanching, if both values are identical only a single value is listed for brevity

^c^Per liter of process volume

^d^Calculated for freshly infiltrated plant material

^e^The depth filtrate was concentrated 20-fold by UF/DF. DM, dry mass; LD_50_, median lethal dose; n.a., not applicable; UF/DF, ultrafiltration/diafiltration

We found that, even assuming LD_50_ values of 1 ng kg^−1^, the process volumes posing an acute threat to operator health when handling bacteria during cloning or cultivation were either in the same order of magnitude as, or even larger than, the total volume present at that stage, or the critical volumes were so large (several milliliters) that an accidental intravenous exposure is implausible. We concluded that the risk for operators handling such bacteria was low despite some toxin expression in bacteria caused by nonspecific promoter activity.

Blanching inactivated bacteria on the infiltrated leaves (and for all subsequent steps) but had no relevant effect on the concentration of toxic protein because the nonspecific expression in *A. tumefaciens* was marginal compared to that intentionally triggered in the plant material (Table 2). Accordingly, toxic protein concentrations were dominated by the transient expression in plants at all process steps after infiltration. As we expected, the toxic protein concentration increased substantially following the UF/DF concentration step, suggesting that the purification process is potentially hazardous (Table 2, Fig. 1). Based on our calculation, only the production of highly potent proteins, such as botulinum toxin with an LD_50_ of ~ 1 ng kg^−1^ [81], can pose a relevant safety risk during downstream processing. In such cases, the corresponding critical volumes were in the microliter range, especially after the UF/DF step, and accidental intravenous exposure to operators therefore becomes plausible. However, these process steps were GMO-free. Therefore, we concluded that the toxic protein accumulating in the plants would pose a relevant safety risk to both operators and the environment, not the small amounts of protein unintentionally produced in the bacteria.

## Conclusions

We detected the nonspecific accumulation of recombinant proteins in *E. coli* at levels of up to 10 µg L^−1^ fermentation broth, but this fell to < 1 µg L^−1^ at the time of cell harvest. The corresponding values were ~ 20-fold lower in *A. tumefaciens*. The critical volumes of process liquids associated with acute toxicity were several milliliters or more assuming typical OD_600nm_ values of 2–10 and LD_50_ values as low as 1 ng kg^−1^ body mass. Accordingly, the unintended intravenous exposure of operators appears unlikely. In contrast, critical volumes during downstream processing, when no GM bacteria are present, were in the microliter range. Furthermore, no airborne distribution of bacteria was detected during infiltration or blanching, indicating that the threat to the environment due to accidental spread from laboratories is negligible. Here, we selected the LPH leader peptide because it facilitates an effective targeting of the secretory pathway where accumulation of the model proteins we used had reported to be highest. Whereas we assume that the accumulation of proteins will be similar when using other targeting signals, we cannot rule out that changes in the signal peptide may also affect expression in bacteria. However, such changes are typically moderate and will not distort the results in a relevant manner. We conclude that nonspecific protein expression in bacteria resulting from residual promoter activity does not pose a relevant risk to experimenters or the environment.

However, some current safety regulations for the risk assessment of GMOs, such as the *E. coli* and *A. tumefaciens* strains we used, appear to use binary decisions (i.e., activity *vs* inactivity of a promoter) instead of quantitative data representing protein accumulation. Accordingly, bacteria containing a vector encoding a toxin under the control of the CaMV35S promoter intended for transient expression in plants would be classified as BSL-2 because residual promoter activity has been shown in the bacteria, even though the data presented here indicate that such activity does not translate into an actual risk for operators or the environment.

Such a binary decision strategy may reduce the workload for regulatory bodies when they assess and rate the risk associated with individual research projects. However, this is likely to reduce scientific innovation, especially in the field of urgently-needed potent new cancer drugs, due to the increased administrative effort and additional infrastructural requirements even for scouting experiments. The application of quantitative rather than qualitative decision criteria for the classification of GMOs would accelerate the development of innovative biopharmaceuticals.

## Supplementary Information

**Additional file 1**. **Table S1**: Promoter sequences used to generate the constructs for promoter activity tests. **Table S2**: Primers used to clone promoter variants. **Table S3**: Plasmids generated for promoter activity tests in *E. coli* and *A. tumefaciens*. **Figure S1**: Sampling positions for air contamination measurements. Distribution of selective and non-selective plates during the blanching processand infiltration processincluding three individual sampling positions.

**Additional file 2**. **Figure S2**: Evaluation of recombinant protein accumulation in plant cell packs. Full size 2 western blots of triplicate PCP extracts using plasmids with different promotersfor the expression of IgG1and DsRed. DsRed was detected using a rabbit anti-His6 4 primary antibody and an alkaline phosphatase-labeled goat anti-rabbit secondary 5 antibody, whereas IgG1 was detected using an AP-labeled goat anti-human antibody. 6 CaMV35S – double-enhanced cauliflower mosaic virus 35S promoter with strong activity in 7 plants; bla – β-lactamase promoter with activity in bacteria; T7 – bacteriophage T7 promoter 8 with minimal activity in bacteria unless the corresponding polymerase is expressed.

## Data Availability

The data that support the findings of this study are available from the corresponding author upon reasonable request.

## Abbreviations

ANOVA: Analysis of variance
AP: Alkaline phosphatase
*bla*: β-Lactamase promoter
BY-2: Bright Yellow 2
CaMV35S: Double enhanced cauliflower mosaic virus 35S promoter
CHS: *Petroselinum hortense* Chalcone synthase
DSP: Downstream processing
ELISA: Enzyme-linked immunosorbent assay
ER: Endoplasmic reticulum
His_6_: 6 × Histidine tag
IgG1: Immunoglobulin subclass G1
LD_50_: Median lethal dose
LPH: Signal peptide, murine antibody heavy chain
OD: Optical density
PCP: Plant cell pack
POI: Protein of interest
SD: Standard deviation
T7: Bacteriophage T7 RNA polymerase promoter
LDS: Lithium dodecyl sulfate
UTR: Untranslated region
YEB: Yeast extract broth
