# Supplementary material for: Risk assessment and bioburden evaluation of Agrobacterium tumefaciens-mediated transient protein expression in plants using the CaMV35S promoter
Source: BMC Biotechnol. 2023 Jun 7;23:14. doi: 10.1186/s12896-023-00782-w (PMC10246419; doi:10.1186/s12896-023-00782-w)
Supplement: Supplementary file 1 — Additional file 1. Table S1: Promoter sequences used to generate the constructs for promoter activity tests. Table S2: Primers used to clone promoter variants. Table S3: Plasmids generated for promoter activity tests in E. coli and A. tumefaciens. Figure S1: Sampling positions for air contamination measurements. Distribution of selective and non-selective plates during the blanching processand infiltration processincluding three individual sampling positions. [file 12896_2023_782_MOESM1_ESM.docx]

**Title: Risk assessment and bioburden evaluation of *Agrobacterium tumefaciens*-mediated transient protein expression in plants using the CaMV35S promoter**

Matthias Knödler^1,2^, Paul Winman Reunious^1,2^, Johannes Felix Buyel^1,2,3*^

^1^ Fraunhofer Institute for Molecular Biology and Applied Ecology IME, Forckenbeckstrasse 6, 52074 Aachen, Germany

^2^ Institute for Molecular Biotechnology, RWTH Aachen University, Worringerweg 1, 52074 Aachen, Germany

^3^ University of Natural Resources and Life Sciences, Vienna (BOKU), Department of Biotechnology (DBT), Institute of Bioprocess Science and Engineering (IBSE), Muthgasse 18, A-1190 Vienna, Austria

*Corresponding author contact information: Tel: +49 241 6085 13162; fax +49 241 6085 10000; e-mail address: johannes.buyel@rwth-aachen.de; ORCID: 0000-0003-2361-143X

**Supplementary materials:**

Table S1: Promoter sequences used to generate the constructs for promoter activity tests.

| Promoter name (nucleotide count) | Promoter nucleotide sequence |
| --- | --- |
| CaMV35S (694) | GGTCCAAAGACCAGAGGGCTATTGAGACTTTTCAACAAAGGGTAATATCGGGAAACCTCCTCGGATTCCATTGCCCAGCTATCTGTCACTTCATCGAAAGGACAGTAGAAAAGGAAGATGGCTTCTACAAATGCCATCATTGCGATAAAGGAAAGGCTATCGTTCAAGATGCCTCTACCGACAGTGGTCCCAAAGATGGACCCCCACCCACGAGGAACATCGTGGAAAAAGAAGACGTTCCAACCACGTCTTCAAAGCAAGTGGATTGATGTGATACATGGTGGAGCACGACACTCTCGTCTACTCCAAGAATATCAAAGATACAGTCTCAGAAGACCAGAGGGCTATTGAGACTTTTCAACAAAGGGTAATATCGGGAAACCTCCTCGGATTCCATTGCCCAGCTATCTGTCACTTCATCGAAAGGACAGTAGAAAAGGAAGATGGCTTCTACAAATGCCATCATTGCGATAAAGGAAAGGCTATCGTTCAAGATGCCTCTACCGACAGTGGTCCCAAAGATGGACCCCCACCCACGAGGAACATCGTGGAAAAAGAAGACGTTCCAACCACGTCTTCAAAGCAAGTGGATTGATGTGATATCTCCACTGACGTAAGGGATGACGCACAATCCCACTATCCTTCGCAAGACCCTTCCTCTATATAAGGAAGTTCATTTCATTTGGAGAGGACA |
| *bla* promoter (92) | TTTGTTTATTTTTCTAAATACATTCAAATATGTATCCGCTCATGAGACAATAA CCCTGATAAATGCTTCAATAATATTGAAAAAGGAAGAGT |
| T7 promoter (23) | TAATACGACTCACTATAGGGAGA |

Table S2: Primers used to clone promoter variants.

| Primer name (length) | Sequence |
| --- | --- |
| bla-frw (39) | CTGCTGGGCGCGCCTTTGTTTATTTTTCTAAATACATTC |
| bla-rvs (36) | CGTTCGGAATTCACTCTTCCTTTTTCAATATTATTG |
| T7-frw (58) | CTAGTCGGCGCGCCTAATACGACTCACTATAGGAATTCGTATTTTTACAACAATTACC |
| T7-rvs (18) | TGAGAAGAGAAGGTGAAG |

Table S3: Plasmids generated for promoter activity tests in E. coli and A. tumefaciens.

| Promoter | 5′-UTR | Signal peptide | Encoded protein | | UniProt ID | C-terminal tag | | Target compartment |
| --- | --- | --- | --- | --- | --- | --- | --- | --- |
| CaMV35S | CHS | LPH/KDEL | DsRed | Q9U6Y8 | | His_6_ | ER | |
| *bla* | CHS | LPH/KDEL | DsRed | Q9U6Y8 | | His_6_ | ER | |
| T7 | CHS | LPH/KDEL | DsRed | Q9U6Y8 | | His_6_ | ER | |
| CaMV35S | Omega | LPH_2_/KDEL | M12 IgG1 | n.a. | | none | ER | |
| *bla* | Omega | LPH_2_/KDEL | M12 IgG1 | n.a. | | none | ER | |
| T7 | Omega | LPH_2_/KDEL | M12 IgG1 | n.a. | | none | ER | |

CaMV35S – double enhanced cauliflower mosaic virus 35S promoter; *bla* – β-lactamase promoter; CHS – *Petroselinum hortense* chalcone synthase; ER – endoplasmic reticulum; His_6_ – 6 × histidine tag; LPH – signal peptide from the heavy chain of the murine tobacco mosaic virus-specific monoclonal antibody 24; n.a. – not applicable; omega – omega sequence of tobacco mosaic virus; T7 – bacteriophage T7 RNA polymerase promoter.


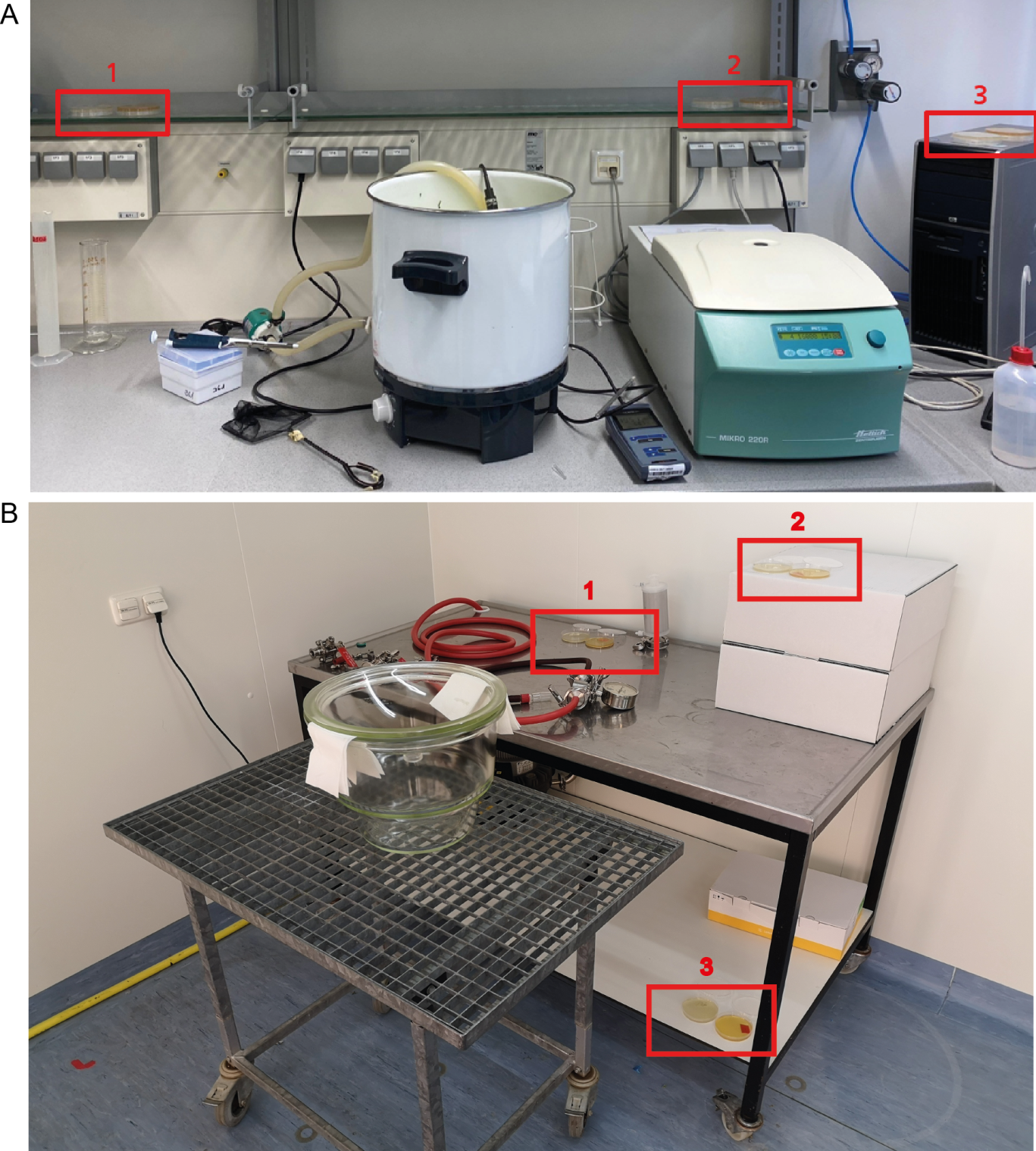


Figure S1: Sampling positions for air contamination measurements. Distribution of selective and non-selective plates during the blanching process (A) and infiltration process (B) including three individual sampling positions (1–3).
