# Supplementary material for: Risk assessment and bioburden evaluation of Agrobacterium tumefaciens-mediated transient protein expression in plants using the CaMV35S promoter
Source: BMC Biotechnol. 2023 Jun 7;23:14. doi: 10.1186/s12896-023-00782-w (PMC10246419; doi:10.1186/s12896-023-00782-w)
Supplement: Supplementary file 2 — Additional file 2. Figure S2: Evaluation of recombinant protein accumulation in plant cell packs. Full size 2 western blots of triplicate PCP extracts using plasmids with different promotersfor the expression of IgG1and DsRed. DsRed was detected using a rabbit anti-His6 4 primary antibody and an alkaline phosphatase-labeled goat anti-rabbit secondary 5 antibody, whereas IgG1 was detected using an AP-labeled goat anti-human antibody. 6 CaMV35S – double-enhanced cauliflower mosaic virus 35S promoter with strong activity in 7 plants; bla – β-lactamase promoter with activity in bacteria; T7 – bacteriophage T7 promoter 8 with minimal activity in bacteria unless the corresponding polymerase is expressed. [file 12896_2023_782_MOESM2_ESM.pdf]

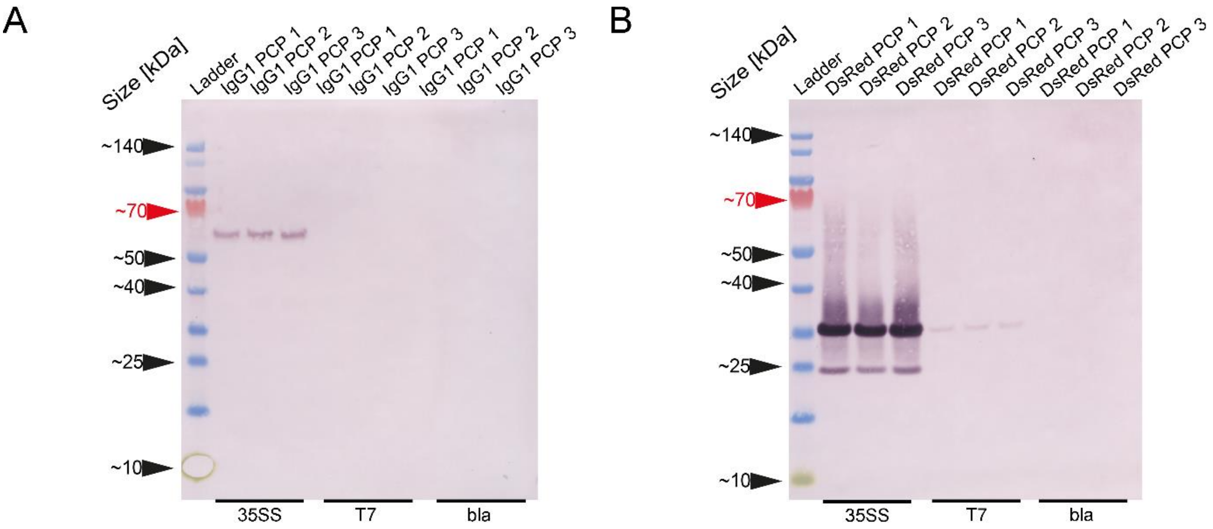

Figure S2: Evaluation of recombinant protein accumulation in plant cell packs (PCPs). Full size western blots of triplicate PCP extracts using plasmids with different promoters (35SS, T7 or bla) for the expression of IgG1 (A) and DsRed (B). DsRed was detected using a rabbit anti-His_6 primary antibody and an alkaline phosphatase (AP)-labeled goat anti-rabbit secondary antibody, whereas IgG1 was detected using an AP-labeled goat anti-human antibody. CaMV35S – double-enhanced cauliflower mosaic virus 35S promoter with strong activity in plants; *bla* – β-lactamase promoter with activity in bacteria; T7 – bacteriophage T7 promoter with minimal activity in bacteria unless the corresponding polymerase is expressed.
